# Comparison of boiling versus magnetic bead techniques in nucleic acid extraction for human papillomavirus detection: evidence based 17,179 cases

**DOI:** 10.1186/s12985-025-02999-x

**Published:** 2025-11-12

**Authors:** Mingjian Bai, Yunxiang Li, Qian Gao, Zhiyong Lv, Fengxian Fu, Guowei Liang, Fucun Ma, Jing Feng

**Affiliations:** 1 Department of Clinical Laboratory, Aerospace Center Hospital, 15 Yuquan Road, Haidian District, Beijing, 100049 P. R. China; 2 Peking University Fifth School of Clinical Medicine, Beijing, 100730 P. R. China; 3 Department of Pathology, Aerospace Center Hospital, Beijing, 100049 P. R. China; 4 Department of Gynecology, Aerospace Center Hospital, Beijing, 100049 P. R. China

**Keywords:** Human papillomavirus, Nucleic acid extraction, Boiling method, Magnetic bead method

## Abstract

**Purpose:**

Present study aimed to compare the boiling and magnetic bead nucleic acid extraction method for HPV genotypes detection.

**Methods:**

By mixing hemoglobin with HPV positive quality control products, the anti-interference ability of two nucleic acid extraction methods on hemoglobin was simulated. Subsequently, on the basis of paired small sample and longitudinal large sample comparisons, the HPV detection rates between the boiling and magnetic bead-based method were evaluated. Finally, the cost-benefit comparisons of the two extraction methods were further evaluated. A two-sided *P* value of less than 0.05 was considered statistically significant.

**Results:**

As for boiling method, when the hemoglobin concentration exceeded 30 g/L, HPV positive control could not be detected, nevertheless, the HPV positive control can still be detected even when the hemoglobin concentration reached 60 g/L for magnetic bead method. In the paired small-scale (639 cases) experiment, results indicated that the positive detection rate of HPV using the magnetic bead method was significantly higher than that of the boiling method, with positive rates of 20.66% and 10.02% (*P* < 0.001), respectively. Additionally, in the longitudinal large-scale analysis (16540 cases) reached the same conclusion. Compared with boiling method, the cost of magnetic bead method increased by 13.14%, however, the detection rate of HPV increased by 106.19%.

**Conclusion:**

Compared to the boiling method, the magnetic bead-based nucleic acid extraction technique exhibited superior anti-interference capabilities and a significant higher detection rate for HPV. Furthermore, it is highly cost-effective. It is anticipated that the magnetic bead method will be fully adopted for HPV detection in place of the boiling method in the future. Of course, more research is needed to verify this conclusion.

## Introduction

Human papillomavirus (HPV) is a significant public health concern globally [1]. A strong association exists between HPV and various cancers, including cervical and oropharyngeal malignancies [2]. It is estimated that 4.5% of all cancers worldwide are attributable to HPV infection, with 8.6% in women and 0.8% in men [3]. HPV infections resulting in considerable psychological and economic burdens for both patients and healthcare systems. Thus, the reliable HPV genotyping detection is important for clinical management decisions [4, 5].

The reported HPV infection rate varied among different studies [6, 7], which may be attributed to several factors. Firstly, the selection of study populations can greatly influence the reported infection rates, some research focuses solely on gynecological patients [8] or health check-up populations [9], while others include both groups [10]. Secondly, disparities in healthcare conditions among different countries can lead to varying HPV infection rates, reflecting the impact of public health initiatives and access to preventive measures [11]. Furthermore, the choice of nucleic acid extraction methods, which is often overlooked, may significantly affect the detection rates of HPV [12]. Different nucleic acid extraction methods can lead to variations in the concentration, purity, and fragment length of the extracted nucleic acids, which in turn can affect the subsequent PCR amplification detection of HPV [13]. Thus, it is critical to explore and refine effective nucleic acid extraction methods for HPV, which hold substantial clinical significance in enhancing early detection and treatment outcomes.

The two commonly applied nucleic acid extraction approaches for detecting HPV include boiling method and magnetic bead method. The present study aimed to compare these two nucleic acid extraction methods in HPV detection. First, due to the special nature of cervical swab specimens, which are often contaminated with blood, such as during menstruation or pathological bleeding. Therefore, this study would compare the anti-interference capabilities of two nucleic acid extraction methods for blood-contaminated specimens. Secondly, present study aimed to compare the ability of two extraction methods to detect HPV in the paired specimens with a small sample size. If the above comparisons could confirm that either the boiling method or the magnetic bead method has better extraction capabilities, the study would further expand the sample size for comparison to further verify which nucleic acid extraction method is more advantageous for HPV detection. Ultimately, the goal was to select a more suitable nucleic acid extraction method for HPV testing and to provide reliable recommendations for clinical application.

## Methods

Present research was approved by IRB of Aerospace Center Hospital in 2022 and conducted in accordance with the declaration of Helsinki.

### Cervical swab samples collection

To ensure the accuracy of the HPV detection results, all female participants were advised to refrain from using vaginal medications and to avoid the menstrual period. During specimen collection, clinical physicians used a speculum to expose the cervix, a cotton swab was applied to wipe away cervical mucus and excess secretions on the surface. The cervical swab was gently rotated clockwise and inserted about 1 cm into the cervical canal. After rotating in the same direction for 5 circles to collect exfoliated cells. The brush was then broken at the neck of the preservation solution bottle and sent to the laboratory for testing as soon as possible.

### Nucleic acid extraction methods

The boiling-based DNA extraction procedure was performed as follows. The specimen tube was shaken thoroughly to mix the contents, and 300 µL sample was transferred into a 1.5 mL Eppendorf (EP) tube. The tube was centrifuged at 14, 000 rpm for 3 min, and the supernatant was discarded. 200 µL of nucleic-acid extraction reagent (mainly consisting of CheLex 100; Tellgen Corporation, China) were added to the pellet and mixed thoroughly. The mixture was incubated in a 100 °C metal bath for 15 min and then centrifuged again at 14, 000 rpm for 5 min. Finally, 5 µL of the supernatant was added to the corresponding PCR reaction tubes.

While the magnetic beads-based DNA extraction (qEx-DNA/RNA virus T183, Tianlong Corporation, China) procedure included: A 300 µL sample was loaded into the extraction plate, and the entire procedure was run automatically on the PANA 9600 s instrument (Tianlong Corporation, China). The automated protocol consisted of four sequential steps: lysis, magnetic attraction, washing, and elution. Finally, 5 µL of the eluate was also transferred into the corresponding PCR reaction tubes.

### HPV-DNA genotype detection process

HPV testing was performed by Tellgenplex^®^ HPV27 DNA genotyping Test system (Tellgen Corporation, China). The experimental procedure including PCR amplification, hybridization, and fluorescence detection (Luminex 200TM, Thermo Fisher) [14]. The totaling detectable HPV including 27 genotypes: low-risk (LR) HPV types (6, 11, 40, 42, 43, 44, 55, 61, 81, 83) and high-risk (HR) HPV types (16, 18, 26, 31, 33, 35, 39, 45, 51, 52, 53, 56, 58, 59, 66, 68, 82). Finally, phycoerythrin fluorescence values greater than 150 considered as a positive result while less than 150 indicated a negative result.

### Anti-hemoglobin interference ability

Hemoglobin (Hb) has been shown to exert a significant inhibitory effect during PCR amplification [15]. The specific experimental design was as follows. An EDTA-anticoagulated whole blood specimen with a hemoglobin level of 120 g/L was collected. It was then diluted with distilled water to concentrations of 120, 100, 80, 60, 40, 30, 20, 16, 12, 8, 4, and 0 g/L. Subsequently, the positive control specimen (including HPV genotypes 16 and 18) was mixed with the diluted whole blood specimens in a 1:1 ratio. Ultimately, diluting the hemoglobin concentration of the HPV positive control to 60, 50, 40, 30, 20, 15, 10, 8, 6, 4, 2, and 0 g/L. The 300 µL mixed specimens underwent parallel nucleic acid extraction using both the boiling and magnetic bead methods. Each sample was tested for HPV-DNA genotyping three times, and phycoerythrin fluorescence values were recorded.

### Comparative DNA extraction method assessment

Small-scale comparison of the paired samples: A total of 639 specimens for HPV test were acquired between December 19, 2024, and January 4, 2025. Both boiling and magnetic beads method were simultaneously applied for each sample and record the HPV results. The Bar Comparison Charts for each HPV subtype was drawn to more intuitively observe the detection capabilities of the two extraction methods. The commonly used method for HPV nucleic acid extraction in the reagent manual is the boiling method. If the results of the above small sample experiment can meet the prerequisites that the magnetic bead-based HPV detection rate is higher than that of the boiling method. Then, present study would conduct the following large-scale sample validation.

Large-scale longitudinal comparison: First, present study would retrospectively collect HPV test results from laboratory information system (LIS) before December 19, 2024. These results would include samples from both gynecological patients and those undergoing health check-ups, with nucleic acids extracted using the boiling method. Subsequently, the study would prospectively collect specimens after January 4, 2025, using magnetic bead extraction method to detect HPV. Finally, the HPV detection rates of the two groups would be compared.

### Statistical analysis

Statistical analyses were conducted using *SPSS* software (version 16.0; IBM Corporation, USA). *Chi-square* tests or *Fisher’s* exact tests were applied when comparing HPV infection rates across different groups, as appropriate. McNemar test was employed to compare the HPV detection rates between boiling and magnetic bead methods within the same population. The consistency between boiling and magnetic bead methods was evaluated using the Kappa value. A Kappa value greater than 0.75 indicates good consistency, a Kappa value between 0.4 and 0.75 indicates moderate consistency, and a Kappa value < 0.40 indicates poor consistency. When comparing phycoerythrin fluorescence values between boiling and magnetic bead method, a *t*-test or non-parametric test was used, as appropriate. Additionally, Microsoft Excel 2019 was used to draw Bar Comparison Charts for the HPV results of the 639 paired samples. Statistical significance was set at *p* < 0.05.

## Results

### Anti-hemoglobin interference comparison

The simulated hemoglobin interference experiment shows that different nucleic acid extraction methods vary in their resistance to interference. For the boiling method, HPV positive control can be detected when hemoglobin concentration is 20 g/L; however, at 30 g/L, hemoglobin strongly interferes, causing negative HPV results. In contrast, even when the hemoglobin concentration reached 60 g/L, the magnetic bead method for nucleic acid extraction remains unaffected, and the HPV positive control can still be detected. Details are shown in Table 1.

**Table 1:** Hemoglobin interference for boiling and magnetic bead methods in detecting HPV

Hb (g/L)	Boiling method						Magnetic bead method					
	HPV-16 Result (value)			HPV-18 Result (value)			HPV-16 Result (value)			HPV-18 Result (value)		
60	N (17.5)	N (11)	N (17.5)	N (18.5)	N (19.5)	N (14.5)	P (558)	P (719.5)	P (757)	P (616.5)	P (709)	P (757)
50	N (15)	N (18)	N (9.5)	N (27)	N (12)	N (12.5)	P (1108)	P (991)	P (1031)	P (1021.5)	P (913.5)	P (959)
40	N (9.5)	N (10)	N (15.5)	N (15)	N (8)	N (7)	P (977)	P (1085)	P (1308)	P (931)	P (1004)	P (1154.5)
30	N (10)	N (5)	N (1)	N (26)	N (9)	N (6)	P (1207)	P (1113)	P (1125)	P (1113)	P (1073)	P (1017)
20	P (1226)	P (1013.5)	P (1379)	P (1508)	P (1233)	P (1469)	P (1047)	P (1311)	P (1304)	P (911)	P (1122.5)	P (1068)
15	P (1266)	P (1234)	P (982)	P (1368)	P (1362)	P (1227)	P (1329)	P (1338)	P (1391.5)	P (1045)	P (1047)	P (1187)
10	P (1544)	P (1445)	P (1235.5)	P (1491)	P (1579.5)	P (1456)	P (1469)	P (1469)	P (1308.5)	P (1270)	P (1257)	P (1101)
8	P (1462.5)	P (1452)	P (1440)	P (1658)	P (1549)	P (1614)	P (1539)	P (1498)	P (1439)	P (1258)	P (1347)	P (1163)
6	P (1537.5)	P (1218)	P (1610)	P (1517)	P (1442)	P (1597)	P (1498)	P (1435)	P (1490)	P (1327.5)	P (1281)	P (1365)
4	P (1657)	P (1334.5)	P (1293)	P (1790)	P (1530.5)	P (1396)	P (1831)	P (1582)	P (1723.5)	P (1368)	P (1360)	P (1339)
2	P (1562)	P (1731)	P (1662.5)	P (1583.5)	P (1925.5)	P (1671)	P (1587)	P (1826)	P (1719.5)	P (1468)	P (1509.5)	P (1509)
0	P (1569)	P (1303)	P (1375)	P (1782.5)	P (1535)	P (1512)	P (1781)	P (1816)	P (1934)	P (1480)	P (1442)	P (1629.5)

N: negative; P: positive. Value: phycoerythrin fluorescence value (*Median*) greater than 150 considered as a positive result and no more than 150 indicated a negative result

### Paired-sample comparison

**Fig. 1:**
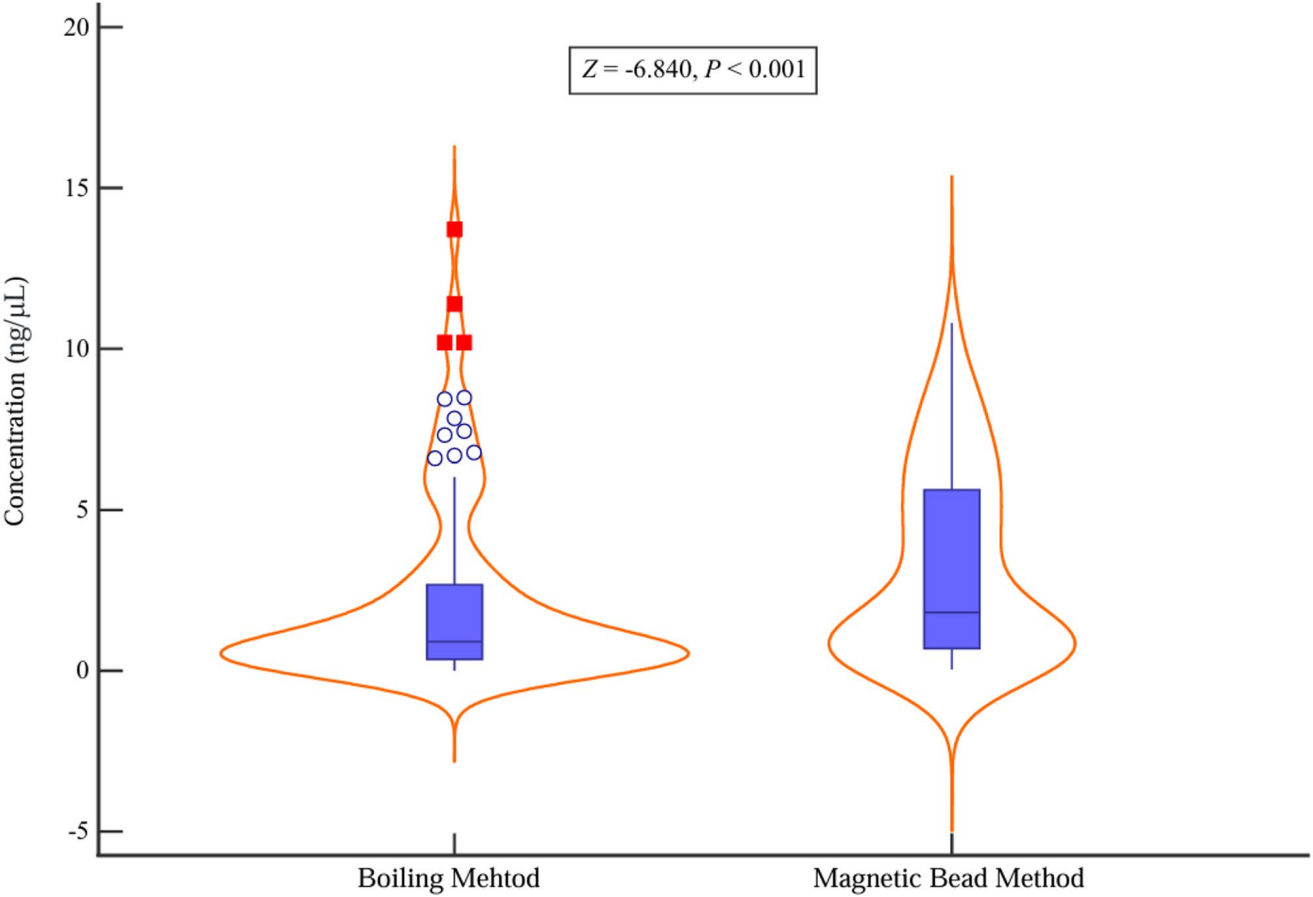
The nucleic acid concentration (ng/µL) between the boiling and magnetic beads method (*P* < 0.001)

A total of 639 cases underwent nucleic acid extraction by both boiling and magnetic bead methods. The nucleic acid concentrations (ng/µL) were 0.91 (0.36, 2.74) for boiling and 1.82 (0.69, 5.63) for magnetic bead methods, respectively (*Z* = −6.840, *P* < 0.001, Wilcoxon test) (Fig. 1). According to Thermo Fisher Scientific’s “Interpretation of Nucleic Acid 260/280 Ratios,” when the nucleic acid concentration is below 10 ng/µL, the OD260/OD280 ratio may be inaccurate. Therefore, this study did not determine the purity of nucleic acids extracted by the two methods. The fluorescence value of globin 28 (served as reference gene) between the two groups were 1537 ± 430 vs. 1440 ± 540, *t* = −1.734, *P* = 0.086 (paired sample *t*-test).

**Fig. 2:**
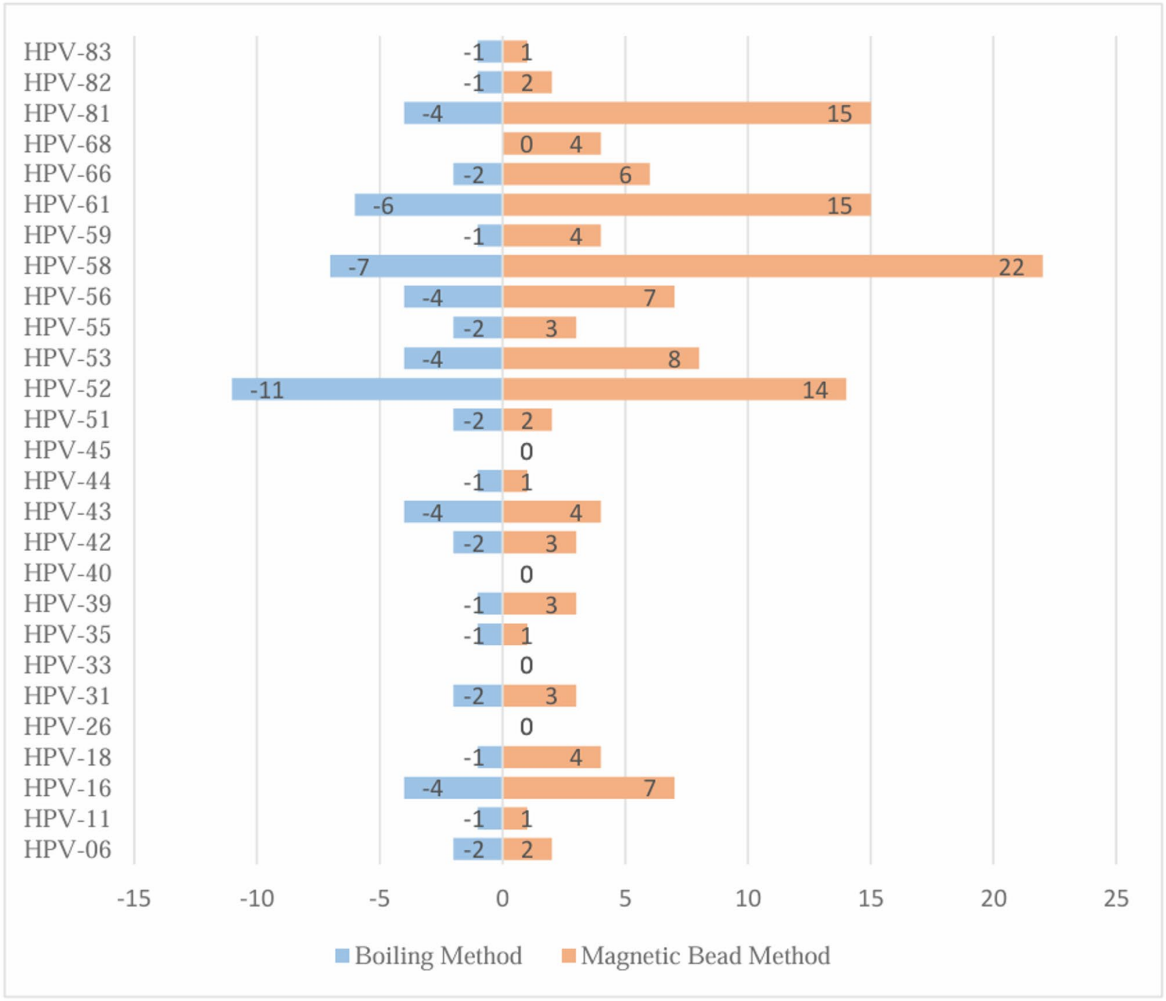
The detailed HPV genotypes displayed between boiling and magnetic bead methods in 639 paired participants

A total of 138 HPV genotypes were detected among the 639 subjects. In detail, there were 64 HPV genotypes detected by the boiling method with a positive rate of 10.02%, while there were 132 HPV genotypes identified by magnetic beads method with the positive rate of 20.66% (Table 2). The *McNemar* test denotes statistical significance between the two methods (*P* < 0.001), and *Kappa* test indicates that the consistency is generally acceptable (*Kappa* = 0.528, *P* = 0.044). For the detection results of each HPV subtype, the Bar Comparison Chart provides a more detailed result display, as shown in Fig. 2. Further, the quantified fluorescent values of the 138 positive HPV genotypes between the two methods were 110 (14, 497) vs. 749 (317, 1280), *Z* = −8.912, *P* < 0.001. respectively.

**Table 2:** The detailed detected HPV genotypes among the 639 paired subjects

Boiling method	Magnetic bead method		Total
	Positive	Negative	
Positive	58	6	64
Negative	74	501	575
Total	132	507	639

### Large-scale longitudinal comparison

From August 29, 2024 to December 19, 2024, a retrospective analysis of 7,532 individuals for HPV testing were conducted from LIS. Among them, 940 were gynecological patients, and 6,592 underwent health check-ups. A total of 861 HPV genotypes positive results were detected, with a positivity rate of 11.43% (861/7,532). The present study with a small sample confirmed that the positive rate of HPV detection after magnetic beads based nucleic acid extraction method was significantly higher than that of the boiling method. Therefore, a total of 9,008 individuals for HPV detection based-on magnetic bead method were prospectively included from January 5, 2025 to May 24, 2025, among which 563 were gynecological patients and 8,445 were health check-up individuals, resulting in the detection of 1,584 positive HPV genotypes, with a positive rate of 17.58% (1,584/9,008), and the difference is statistically significant (*χ*^*2*^ = 123.289, *P* < 0.001). In addition, the proportion of gynecological and health check-up personnel was compared between the two groups, and it was found that the proportion of gynecological personnel in retrospective studies (12.48%) was higher than that in prospective studies (6.25%), with a statistically significant difference (*χ*^*2*^ = 192.711, *P* < 0.001). The median ages of the two groups were 42 (36, 54) vs. 42 (36, 51), respectively (*Z* =−4.118, *P* < 0.001). Table 3.

**Table 3:** Large-scale longitudinal comparison HPV detection rates between boiling and magnetic bead method

	Positive	Negative	Total
Boiling method	861	6671	7532
Magnetic bead method	1584	7424	9008
Total	2445	14,095	16,540

### Cost-benefit comparisons

Based on the results of 639 paired cases, a cost-benefit comparison was performed. The basic testing cost of the HPV detection kit was 137 CNY in present study, and the boiling method for nucleic acid extraction reagent was supplied free of charge. The cost of the magnetic bead nucleic acid extraction reagent per person was 18 CNY. Therefore, if the magnetic bead method is adopted to extract nucleic acid for HPV testing, the cost would increase by 13.14% (18/137). Correspondingly, compared with the original boiling method, the HPV detection rate based on the magnetic bead method increased by 106.19%: (20.66−10.02)/10.02 × 100%.

## Discussion

The current study demonstrated that the magnetic bead method exhibited a significantly greater capacity to counteract hemoglobin interference compared to the boiling method. In both paired small sample and longitudinal large sample comparisons, the magnetic bead-based nucleic acids extract method significantly increased the HPV detection rate. The HPV detection rate using the magnetic bead method has greatly increased with only a minimal cost increase, demonstrating excellent cost-effectiveness.

In clinical practice, nucleic acid extraction is an important step in HPV testing. The interference of hemoglobin is particularly significant in nucleic acid extraction process [16]. Studies have shown that when hemoglobin is present in the sample, the traditional boiling method may not effectively extract high purity nucleic acid [17]. This study found that the magnetic bead method was able to detect HPV genotypes even the hemoglobin at a concentration of 60 g/L, while the boiling method failed to detect HPV when the hemoglobin concentration at 30 g/L. Hence, the magnetic bead method has a significantly stronger ability to resist hemoglobin interference compared to the boiling method. Firstly, iron ions within hemoglobin generate reactive oxygen species through Fenton reactions, which leads to DNA strand breaks and nucleic acid degradation. Secondly, hemoglobin molecules can directly bind to DNA polymerase, thereby inhibiting its catalytic activity. Lastly, the porphyrin ring of heme can intercalate with the DNA double helix, obstructing the binding of primers to the template. To counteract this inhibitory effect, silica membrane adsorption columns or magnetic bead techniques is recommended to remove hemoglobin [18]. This is also the reason why when collecting HPV specimens, care is taken to avoid menstruation and significant blood contamination, and if necessary, saline is used for rinsing before collecting cervical swabs [19]. In summary, when encountering blood-contaminated specimens in clinical settings, the magnetic bead method demonstrates stronger anti-interference capabilities.

Previous research indicated that the quality of nucleic acid extraction directly influences the sensitivity and specificity of downstream PCR assays [20], however, systematic comparisons between these two methods in the context of HPV detection are limited. To be cautious, this study first conducted a parallel comparison experiment with a small sample of 639 cases. Compared to the boiling method, the HPV detection rate significantly increased with the magnetic bead method. Especially, there were 74 cases that were positive by the magnetic bead method but negative by the boiling method, accounting for as high as 11.58%. Nevertheless, there were only 6 cases positive by the boiling method and negative by the magnetic bead method, accounting for only 0.94%. The results of present study suggest that if the magnetic bead method is used to replace the boiling method, it is very likely to increase the detection rate, and the chance of missed detection is very small. Subsequently, the large longitudinal cohort sample also confirmed this conclusion, which showing that the positive rate of HPV detection using the magnetic bead method was significantly higher than that of the boiling method. Unlike the small sample, the patient source ratio for the boiling method and the magnetic bead method in this study was different, with a higher proportion of gynecological patients in the boiling method group. Generally, this segment of the population has a significantly higher positive rate of HPV compared to the health check-up population [21–23], but the positive rate is still lower than that of the magnetic bead method. Therefore, the magnetic bead-based nucleic acid extraction method significantly improves the detection rate of HPV.

The reason for concluding that the magnetic bead method has a higher HPV detection rate is determined by the principles of different methods for nucleic acid extraction. The magnetic bead method has the following important advantages. First, magnetic bead-based extraction method has been shown to produce higher yields of nucleic acids, making them particularly suitable for samples with low copy numbers [24]. Present study also confirms this: in the paired small sample research, the nucleic acid concentration extracted by the magnetic bead method was approximately twice that obtained by the boiling method. Furthermore, in 138 positive specimens, the fluorescence value of HPV using the magnetic bead method was also significantly higher than that of the boiling method. Second, due to the low nucleic acid concentration extracted in this study, it is not possible to perform purity verification based on the present conditions. However, most studies have confirmed that the purity of nucleic acid extraction using the magnetic bead method is significantly higher than that of the boiling method, which possess the ability to achieve high nucleic acid purity and effectively remove inhibitors such as hemoglobin present in biological samples [25–27]. Finally, the magnetic bead method automated integrating lysis, binding, washing, and elution [28], which could reduce human error, increase throughput, standardize of protocols, and adaptability to various sample types [29, 30]. In all, the magnetic bead method owns the advantages of extraction efficiency, nucleic acid purity, and potential for automation. Nevertheless, why for 6 cases the boiling method could detect HPV but the magnetic method failed? After carefully reading the instruction manual of this nucleic acid extraction reagent (T183), we found that this nucleic acid extraction reagent without proteinase K. The absence of proteinase K can lead to protein residue and downstream PCR inhibition. Therefore, this reagent is still not ideal in present research. In the future, the new reagent which is particularly suitable for HPV nucleic acid extraction will be employed, and the detection rate of HPV might increase further.

Recent literature shows that HPV nucleic acid extraction methods mainly use the magnetic bead method and are mostly applied in developed countries [31–34]. So, why do some developing countries or regions still use the boiling method? The main reason is for resource constraints or financial burdens. The simplicity and low cost of the boiling method make it a viable option for laboratories lacking advanced extraction technology or for field applications with limited resources [24, 35]. Present study found that the cost increase after adopting the magnetic bead method was only 13.14%, while the detection rate of HPV increased by more than 100%. Therefore, it has an extremely high cost-performance ratio. Of course, the magnetic bead method often requires dedicated nucleic acid extraction instruments, which is another limitation. With the development of the global economy, future investments in healthcare would increase, and at the same time, the cost of nucleic acid extraction using magnetic bead methods would decrease based on rapid technological advancements [36, 37]. Combined with the statistical results of present study, it indicates that replacement of boiling methods by magnetic-bead protocols in HPV genotyping has become an inevitable trend.

This study has the following shortcomings: First, the research only assessed the interference resistance of the boiling method and magnetic bead method with respect to hemoglobin, without evaluating other interfering substances. Second, in the paired comparison, only 639 cases were included due to cost considerations, and the limited small sample size resulted in a limited number of detected HPV subtypes, leading to some HPV subtypes having a count of zero in the Bar Comparison Chart. Third, during the large sample validation, the included populations were different, with varying ratios of gynecological patients to health check populations, which may affect the inference of whether there is a difference in HPV detection rates between the two nucleic acid extraction methods. Future studies need to evaluate the impact of the two methods on HPV detection rates with a larger paired sample, especially covering all HPV subtypes to achieve a comprehensive assessment for each HPV subtype.

## Conclusion

In conclusion, effective management of current HPV infections depends on reliable nucleic acid testing and genotyping. Magnetic bead-based nucleic acid extraction has stronger anti-hemoglobin interference ability, higher detection throughput, higher nucleic acid extraction efficiency, and higher automation compatibility. Furthermore, the magnetic bead method proved highly cost-effective. Consequently, the boiling method is expected to be gradually replaced by the magnetic bead method, which is likely to be widely adopted in HPV testing. However, further validation with larger sample sizes and all HPV subtypes is needed, especially for paired comparisons of the detection capabilities of both methods.

## Data Availability

The datasets used and analyzed during the current study are available from the corresponding author on reasonable request.

## Abbreviations

HPV: Human papillomavirus
LR: Low risk
HR: High risk
Hb: Hemoglobin
LIS: Laboratory information system

## References

[1] Duan LL, Yin H, Li Q, et al. Correlation between human papillomavirus infection and reproduction[J]. Ginekol Pol. 2022;93(4):329–33. 35419800 10.5603/GP.a2021.0175

[2] Kombe KA, Li B, Zahid A, et al. Epidemiology and burden of human papillomavirus and related Diseases, molecular Pathogenesis, and vaccine Evaluation[J]. Front Public Health. 2020;8:552028. 33553082 10.3389/fpubh.2020.552028 PMC7855977

[3] Williams J, Kostiuk M, Biron VL. Molecular detection methods in HPV-related cancers. Front Oncol. 2022;12:864820. 35574396 10.3389/fonc.2022.864820 PMC9092940

[4] Serrano B, Brotons M, Bosch FX, et al. Epidemiology and burden of HPV-related disease. Best Pract Res Clin Obstet Gynaecol. 2018;47:14–26. 29037457 10.1016/j.bpobgyn.2017.08.006

[5] Roberts CC, Swoyer R, Bryan JT, et al. Comparison of real-time multiplex human papillomavirus (HPV) PCR assays with the linear array HPV genotyping PCR assay and influence of DNA extraction method on HPV detection[J]. J Clin Microbiol. 2011;49(5):1899–906. 21346041 10.1128/JCM.00235-10 PMC3122643

[6] Zhou HL, Zhang W, Zhang CJ, et al. Prevalence and distribution of human papillomavirus genotypes in Chinese women between 1991 and 2016: A systematic review[J]. J Infect. 2018;76(6):522–8. 29477803 10.1016/j.jinf.2018.02.008

[7] Ruan Y, Li H, Liu M, et al. A retrospective analysis of human papillomavirus (HPV) prevalence and genotype distribution among 25,238 women in Shanghai, China revealed the limitations of current HPV-based screening and HPV vaccine[J]. Cancer Epidemiol. 2023;84:102372. 37119603 10.1016/j.canep.2023.102372

[8] Ma X, Wu C, Wu T, et al. Genotypic analysis of human papillomavirus in cervical exfoliated cells from women in Zigong[J]. Virol J. 2025;22(1):40. 39962540 10.1186/s12985-025-02652-7 PMC11831773

[9] Gao D, Zhao G, Wang X, et al. Association between high-risk human papillomavirus infection and cervical cytology in health check-up women – 23 PLADs, China, 2023[J]. China CDC Wkly. 2025;7(10):327–33. 40225780 10.46234/ccdcw2025.053 PMC11982919

[10] Wei X, Lu Q, Wang S. Prevalence characteristics of cervical human papillomavirus genotypes in Nanning, China: a 10-year survey of 77,756 women from one medical center. J Med Virol. 2022;94(6):2787–95. 34859449 10.1002/jmv.27498

[11] Spayne J, Hesketh T. Estimate of global human papillomavirus vaccination coverage: analysis of country-level indicators. BMJ Open. 2021;11(9):e52016. 10.1136/bmjopen-2021-052016 PMC8413939 34475188

[12] D’Souza G, Sugar E, Ruby W, et al. Analysis of the effect of DNA purification on detection of human papillomavirus in oral rinse samples by PCR[J]. J Clin Microbiol. 2005;43(11):5526–35. 16272481 10.1128/JCM.43.11.5526-5535.2005 PMC1287828

[13] Shin S, Kim J, Song E, et al. Analytical techniques for nucleic acid and protein detection with single-molecule sensitivity. Exp Mol Med. 2025. 10.1038/s12276-025-01453-w. 40307572 10.1038/s12276-025-01453-w PMC12130535

[14] Liao G, Jiang X, She B et al. Multi-Infection patterns and Co-infection preference of 27 human papillomavirus types among 137,943 gynecological outpatients across China. Front Oncol. 2020;10:1–9. 32318343 10.3389/fonc.2020.00449 PMC7154087

[15] Sidstedt M, Hedman J, Romsos EL, et al. Inhibition mechanisms of hemoglobin, Immunoglobulin G, and whole blood in digital and real-time PCR. Anal Bioanal Chem. 2018;410(10):2569–83. 29504082 10.1007/s00216-018-0931-z PMC5857286

[16] Bae MJ, Lee YM, Choi YS, et al. Simple electric device to isolate nucleic acids from whole blood optimized for point of care testing of brain damage. Curr Neurovasc Res. 2022;19(3):333–43. 36056832 10.2174/1567202619666220903105805 PMC10009893

[17] Wen J, Guillo C, Ferrance JP, et al. Microfluidic chip-based protein capture from human whole blood using octadecyl (C18) silica beads for nucleic acid analysis from large volume samples. J Chromatogr A. 2007;1171(1–2):29–36. 17935724 10.1016/j.chroma.2007.09.057

[18] Kermekchiev MB, Kirilova LI, Vail EE, et al. Mutants of Taq DNA polymerase resistant to PCR inhibitors allow DNA amplification from whole blood and crude soil samples. Nucleic Acids Res. 2009;37(5):e40. 19208643 10.1093/nar/gkn1055 PMC2655666

[19] Chen CJ, Hong MK, Ding DC. Effective reduction in inadequate pap smears by using a saline-lubricated speculum and two glass slides. Taiwan J Obstet Gynecol. 2020;59(6):906–9. 33218410 10.1016/j.tjog.2020.09.018

[20] Kann S, Zabala-Monterroza W, Garcia C, et al. Comparison of the influence of different nucleic acid extraction assays on the sensitivity of *Trypanosoma cruzi*-specific real-time PCR. Microorganisms. 2022;10(8):1554. 10.3390/microorganisms10081554 36013972 10.3390/microorganisms10081554 PMC9414588

[21] Masdoua N, Boublenza L, Hassaine H, et al. Characteristics of HPV infection in women at risk in Western Algeria. Med Mal Infect. 2017;47(1):38–41. 27765475 10.1016/j.medmal.2016.09.002

[22] Yan X, Shen L, Xiao Y, et al. Prevalence, characteristics, and distribution of HPV genotypes in women from Zhejiang Province, 2016–2020. Virol J. 2021;18(1):208. 34670576 10.1186/s12985-021-01676-z PMC8527678

[23] Rui Q, Zhu X, Xu G. Epidemiological analysis of HPV infection in Zhangjiagang, southern Jiangsu province of China: a cross-sectional study. Int J Microbiol. 2025;2025:5576260. 40290128 10.1155/ijm/5576260 PMC12033064

[24] Hongjaisee S, Jabjainai Y, Sakset S, et al. Comparison of simple RNA extraction methods for molecular diagnosis of hepatitis C virus in plasma. Diagnostics. 2022;12(7):1599. 10.3390/diagnostics12071599 35885505 10.3390/diagnostics12071599 PMC9322174

[25] Byrne RL, Cocker D, Alyayyoussi G, et al. A novel, magnetic bead-based extraction method for the isolation of antimicrobial resistance genes with a case study in river water in Malawi. J Appl Microbiol. 2022;133(5):3191–200. 35946113 10.1111/jam.15755 PMC9804433

[26] Fan Y, Dai R, Guan X, et al. Rapid automatic nucleic acid purification system based on gas-liquid immiscible phase[J]. J Sep Sci. 2023;46(6):e2200801. 36661136 10.1002/jssc.202200801

[27] Nie Y, Li X, Yang W, et al. Concanavalin-A-assisted extraction-free one-pot RPA-CRISPR/Cas12a assay for rapid detection of HPV16[J]. Mikrochim Acta. 2025;192(6):354. 40369306 10.1007/s00604-025-07198-7

[28] Cho B, Lee SH, Song J, et al. Nanophotonic cell lysis and polymerase chain reaction with gravity-driven cell enrichment for rapid detection of pathogens. ACS Nano. 2019;13(12):13866–74. 31756079 10.1021/acsnano.9b04685

[29] Wang Y, Huang Y, Peng Y, et al. Development and validation of a rapid five-minute nucleic acid extraction method for respiratory viruses. Virol J. 2024;21(1):189. 39155366 10.1186/s12985-024-02381-3 PMC11331601

[30] Hin S, Paust N, Rombach M, et al. Magnetophoresis in centrifugal microfluidics at continuous rotation for nucleic acid extraction. Micromachines (Basel). 2022;13(12):2112. 10.3390/mi13122112 36557411 10.3390/mi13122112 PMC9787563

[31] Naegele K, Weissbach FH, Leuzinger K, et al. Impact of nucleic acid extraction procedures on human papillomavirus (HPV) detection and genotyping. J Med Virol. 2023;95(2):e28583. 36794677 10.1002/jmv.28583

[32] Bell M, Verberckmoes B, Devolder J, et al. Comparison between the Roche Cobas 4800 human papillomavirus (HPV), Abbott realtime High-Risk HPV, seegene anyplex II HPV28, and novel seegene allplex HPV28 assays for High-Risk HPV detection and genotyping in mocked Self-Samples[J]. Microbiol Spectr. 2023;11(4):e8123. 10.1128/spectrum.00081-23 PMC10433804 37284753

[33] Naegele K, Bubendorf L, Hirsch HH, et al. Comparative evaluation of anyplex II HPV28 and allplex HPV28 molecular assays for human papillomavirus detection and genotyping in anogenital cancer screening. J Med Virol. 2024;96(6):e29649. 38812416 10.1002/jmv.29649

[34] Atchison S, Shilling H, Balgovind P, et al. Evaluation of the Roche magna pure 96 nucleic acid extraction platform for the seegene anyplex II HPV28 detection assay. J Appl Microbiol. 2021;131(5):2592–9. 33942451 10.1111/jam.15126

[35] Boza JM, Manning JC, Erickson DC. Comparison and optimization of simple DNA extraction methods for LAMP-based point-of-care applications employing submillimeter skin biopsies. ACS Omega. 2024;9(37):38855–63. 39310140 10.1021/acsomega.4c05025 PMC11411550

[36] Vutukuru MR, Sharma DK, Chakraborty I, et al. A rapid and high-yield method for nucleic acid extraction. Sci Rep. 2025;15(1):12479. 40216842 10.1038/s41598-025-95226-0 PMC11992011

[37] Pearlman SI, Leelawong M, Richardson KA, et al. Correction to low-resource nucleic acid extraction method enabled by high-gradient magnetic separation. ACS Appl Mater Interfaces. 2024;16(1):1953. 38126871 10.1021/acsami.3c18419 PMC11027509

